# Analysis of Physician Compensation Studies by Gender, Race, and Ethnicity

**DOI:** 10.1089/heq.2021.0098

**Published:** 2022-02-01

**Authors:** Allison R. Larson, Meridith J. Englander, Quentin R. Youmans, Monica Verduzco-Gutierrez, Fatima Cody Stanford, Sheritta A. Strong, Howard Y. Liu, Julie K. Silver

**Affiliations:** ^1^ Department of Dermatology, Georgetown University Medical Center and MedStar Health, Washington, DC, USA.; ^2^ Department of Radiology, Albany Medical College, Albany, New York, USA.; ^3^ Division of Cardiology, Department of Medicine, Northwestern University Feinberg School of Medicine, Chicago, Illinois, USA.; ^4^ Department of Rehabilitation Medicine, Joe R. and Teresa Lozano Long School of Medicine at UT San Antonio, San Antonio, Texas, USA.; ^5^ Massachusetts General Hospital, MGH Weight Center, Department of Medicine-Division of Endocrinology-Neuroendocrine, Department of Pediatrics-Division of Endocrinology, Nutrition Obesity Research Center at Harvard (NORCH), Boston, Massachusetts, USA.; ^6^ Department of Psychiatry, University of Nebraska Medical Center, Omaha, Nebraska, USA.; ^7^ Department of Physical Medicine and Rehabilitation, Harvard Medical School, Massachusetts General Hospital, Spaulding Rehabilitation Hospital, Brigham and Women's Hospital, Boston, Massachusetts, USA.

**Keywords:** ethnicity, gender, physician compensation, physician salary

## Abstract

**Purpose:**

This report investigated physician compensation studies by gender, race, and ethnicity.

**Methods:**

Published U.S. physician compensation studies were assessed.

**Results:**

Of the 47 data sets within 46 studies, 36 analyzed compensation by gender and 32 (88.9%) found disparities. Thirteen and eight analyzed for race and ethnicity, with disparities found in four (30.8%) and none, respectively. The sample sizes of the four data sets with differences by race were among the largest in the subset.

**Conclusion:**

Most studies demonstrate pay disparities for women, but not for people who identify with underrepresented race/ethnic groups; however, small sample sizes may affect results.

## Introduction

Among the most important issues confronting the increasingly diverse physician workforce is fair pay. In the United States, both federal law and many states' laws support compensation for people based on the work that is done rather than who is doing the work.^1^ Despite this, there is a large body of evidence that demonstrates pay gaps for women in general. A recent systematic review found that across countries and medical specialties, women physicians earned significantly less than men despite similar demographic and work-related profiles.^2^

In this review, the pay disparities were often tens of thousands of dollars less annually, which can translate into millions of dollars in lost income and investments throughout one's career.^3^ Less is known about compensation disparities for physicians who identify with racial or ethnic minority groups; however, large surveys such as Medscape^4^ suggest that disparities exist for people who identify with these groups. In this report, we analyzed physician compensation studies published in medical journals to determine what is known about pay disparities as they relate to gender, race, and ethnicity.

## Methods

We searched PubMed on July 1, 2020, for studies on physician compensation published between January 1, 2013, and June 30, 2020. We included studies if they used terms in the title or abstract: “salary” or “compensation” or “wage” or “payment” or “research support” and the term(s) “physician” or “faculty.” We excluded studies that did not include U.S. physician compensation, were not in English, were secondary sources (e.g., reviews, perspectives) that did not present novel data, and studies that focused on Medicare payments only or supplementary income (e.g., industry payments, grant awards). IRB approval was not required as all data collected were publicly available.

In a second round of review, we excluded studies that presented data reported as a percentage (percent funding or percent effort) or a partial component of compensation (not total compensation/salary) or billing metrics (e.g., relative value units).

Two authors (A.R.L. and M.J.E.) independently reviewed the 4,563 articles for inclusion and came to consensus on 62 studies that met the initial criteria. Next, two authors (A.R.L. and Q.R.Y.) independently verified the initial inclusion criteria as well as checked for numeric data on total salary/compensation and came to consensus on 46 studies that met the full inclusion criteria, which included an analysis of 47 data sets in total (one study analyzed two data sets separately). We further evaluated each of the 47 data sets to determine specifics of the analyses and findings of disparities by gender, race, and ethnicity.

## Results

**FIG. 1.**
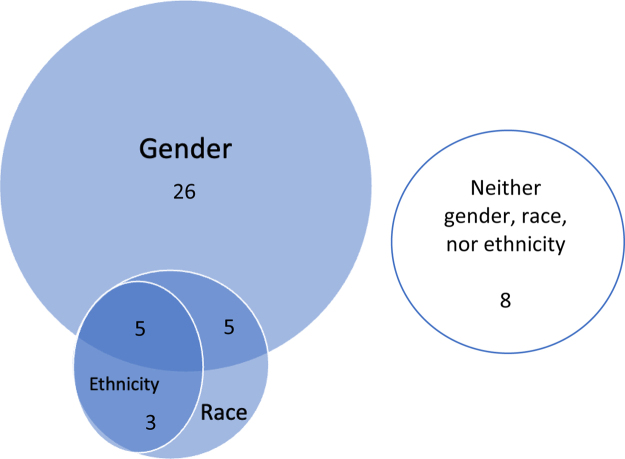
Of the 47 data sets, 26 analyzed by gender only, 3 by ethnicity and race, 5 by gender and race, and 5 by gender, race, and ethnicity. Eight analyzed none of these.

Twelve data sets conducted a multivariable analysis considering at least gender and race and, in some instances, ethnicity. Three of these studies included race/ethnicity in their multivariable model, but did not consider the impact of these terms on compensation separately. These 12 are reported in the first section of Table 1. One study considered the impact of gender and race on compensation individually (nonmultivariable model). Three studies analyzed ethnicity and race, but not gender, and, of these, two used multivariable models adjusting for covariates. Twenty-three studies collected and analyzed data on gender, but not race or ethnicity. Eight studies did not analyze physician compensation data by gender or race or ethnicity (Table 1 and Fig. 1).

**Table 1.** Physician Compensation Studies and Analysis by Gender, Race, and Ethnicity

	Study references First author name (year)	No. of participants	Population studied	Variables in multivariable analysis	Gender disparities studied/found	Race disparities studied/found	Ethnicity disparities studied/found
Multivariable analysis including race/ethnicity/gender (intersection)	Hayes (2020)^15^	2,845	Physicians at one academic institution (Mayo Clinic) with three locations	Gender, race/ethnicity, specialty, leadership position, full-time equivalent status, experience, age, work location, licensure, other compensable activities	Y/N	Y/N	Y/N
	Lo Sasso (2020)^6^	16,047	New York State physicians entering first year of attending-level patient care practice	Specialty training, number of job offers, sex, age, gender, race/ethnicity, citizenship, education and training, educational debt, principal practice setting, location type, obligation to health professional shortage area, weekly patient care hours	Y/Y After adjustment, women made $7,700 less in 1999, rising to $20,200 less by 2017	N/N	N/N
	Langer (2019)^20^	41,396	Physicians in clinical practice who participated in the Community Tracking Survey	Gender, age, degree, training, work hours, weeks worked, revenue sources, practice ownership status, geographic region, metropolitan statistical area category, race, ethnicity	Y/Y Significant differences in gender found by specialty	Y/Y Significant differences in race found by specialty	Y/N
	Pallant (2019)^21^	149	Program director members of the Association of Pediatric Program Directors	Gender, race/ethnicity, age, academic rank, clinical appointment, number of raises, tenure track, years in program director role, number of noncombined residents in program	Y/Y 26.9% of men vs. 6.1% of women earned more than $250,000 annually	Y/N	Y/N
	Apaydin (2018)^11^	439	Physicians from 30 diverse practices within six states	Hours worked, composition of work hours, percent procedural time, specialty, compensation type, age, years in practice, gender, race, ethnicity, state and practice random effects	Y/Y After adjustment, women made $27,404 less income	N/N	N/N
	Read (2018)^22^	374	Members (nonstudent) of the Internal Medicine Insider Research Panel within the American College of Physicians	Bivariate analysis performed comparing salary by gender and one other factor: specialty, employment status, age, race, primary professional setting, primary professional activity, marital status, spousal employment status, parental status	Y/Y Median annual salary for women was $50,000 lower	Y/N	N/N
	Madsen (2017)^19^	1,371	Full-time faculty members in U.S. academic emergency departments via the 2015 Academy of Administrators in Academic Emergency Medicine Salary Survey	Race/ethnicity, region, rank, years of experience, clinical hours, core faculty status, administrative roles, board certification, fellowship training, gender	Y/Y After adjustment, women's salaries were $19,418 lower	Y/N	Y/N
	Freund (2016)^10^	490	Sample of academic medical faculty from 24 U.S. medical schools	Race/ethnicity (combined category), gender, years since first academic appointment, retention in academic career, academic rank, departmental affiliation, percent effort in various areas, marital status, parental status, any leave or part-time status in the years between surveys	Y/Y After adjustment, women earned $16,982 less in annual compensation	Y/N	Y/N
	Ly (2016)^12^	61,327 from ACS survey 17,583 in HSC survey	2000–2013 ACS to 2000–2008 HSC	ACS: age, sex, race, weekly hours worked, state of residence, time period HSC: Age, sex, race, number of hours worked per week, years in practice, practice type, percentage revenue from Medicare or Medicaid, specialty type	Y/Y In both studies, women had lower incomes than men Y/Y	Y/Y In both studies, Black men had lower incomes than White men; incomes were similar for Black and White women Y/Y	N/N N/N
	Jagsi (2013)^8^	1,012	Recipients of NIH mentored career development awards	Gender, age, race, marital status, parental status, additional doctoral degree, academic rank, years on faculty, specialty, institution type, region, institution NIH funding rank, K award type, K award funding institute, K award year, work hours, research time	Y/Y After adjustment, women had lower annual salaries by $10,921	Y/N	N/N
	Seabury (2013)^23^	7,653	1987–2010 March Current Population Survey	Hours worked, age, sex, race, state	Y/Y The annual earnings gap did not change significantly over time ($33,840 in 1987–1990 and $34,620 in 1996–2000)	N/N	N/N
Analysis by race/gender (separate)	Rosenthal (2017)^24^	157	Members of the Academy of Psychosomatic Medicine	Multivariable analysis not performed	Y/Y Average women's salary was $20,000 less	Y/N	N/N
Analysis by race and/or ethnicity only (not gender)	Marcelin (2019)^16^	2,075	Members of the Infectious Diseases Society of North America	Practice type, race, ethnicity	N/N	Y/Y African American ID physicians were paid 7–13% less for most types of employment	Y/N
	Kaplan (2018)^25^	604	Sample of academic medical faculty from 24 U.S. medical schools	Race/ethnicity, setting, rank, effort distribution in teaching, clinical and research activities	N/N	Y/N	Y/N
	Lin (2016)^26^	26 in 2004, 38 in 2009, 54 in 2014	Faculty at one academic (Johns Hopkins) otolaryngology program	Multivariable analysis not performed	N/N	Y/N	Y/N
Analysis by gender only (not race or ethnicity)	Cheng (2020)^27^	72	Members of the American Medical Informatics Association	Multivariable analysis not performed on the physician subset	Y/Y Unadjusted physician salaries were $23,135 lower for women	N/N	N/N
	Gambhir (2021)^28^	170	Surgeons within a large multi-institutional health care system (University of California)	Academic rank, surgical subspecialty, gender	Y/Y Adjusted mean salaries were $45,904 lower for women	N/N	N/N
	Pelley (2020)^29^	Number not given	Data derived from Doximity 2015 average salary numbers by specialty	Specialty, gender	Y/Y Gender composition explained 64% of the variation in salaries among specialties	N/N	N/N
	Sangji (2020)^30^	461	Trauma surgeons, members of The Eastern Association for the Surgery of Trauma	Gender and age or practice type (analyzed separately)	Y/Y Fewer women than men made an income of $300,000 or more (57% vs. 83%)	N/N	N/N
	Shah (2020)^31^	366	Neurocritical care physicians, members of the Neurocritical Care Society	Multivariable analysis not performed	Y/Y Men's median salary range was $276,000–$300,000 compared to women $251,000–$275,000	N/N	N/N
	Winkelman (2020)^32^	85	Urogynecologists employed at public universities with publicly available salary data	Academic rank, leadership roles, years since residency, gender	Y/Y After adjustment, women made on average $37,955 less annually	N/N	N/N
	Dermody (2019)^14^	260	Otolaryngologists employed at Veterans Affairs Medical Centers with level 1 complexity	Number of years since graduation, h-index, gender, geographic location, faculty rank	Y/N	N/N	N/N
	Horowitz (2019)^33^	366	Neonatologists, members of the American Academy of Pediatrics Section on Neonatal-Perinatal Medicine	Gender, geographic region, work with physician assistants, in-house call, years postfellowship, administrative time, daily rounding on critical care patients, clinical time, medical education time, work with neonatal hospitalists, eligibility for annual bonus, large central metropolitan county, academic institution	Y/Y Women's salaries were 3.68% lower in a multivariable model	N/N	N/N
	Wiler (2019)^34^	7,102	Physicians belonging to academic emergency medicine departments	Gender, academic rank, geographic region, type of hospital, years at faculty appointment, year of survey	Y/Y In an adjusted model, women made significantly less than men	N/N	N/N
	Burns (2018)^35^	97	Tenure-track faculty on one academic pathology department (Johns Hopkins)	Type of appointment, academic rank, years at rank, gender	Y/N	N/N	N/N
	Hoops (2018)^36^	86	Surgeons at a single academic institution (Oregon Health & Science University)	Rank, fiscal year, gender	Y/Y Women were compensated significantly less than men; this improved after a compensation plan	N/N	N/N
	Morris (2018)^37^	44	Surgeons at a single academic medical institution (University of Alabama at Birmingham)	Multivariable analysis not performed	Y/Y Women were compensated significantly less than men despite similar RVUs; this improved after a compensation plan	N/N	N/N
	Trotman (2018)^38^	2504	Members of the Infectious Diseases Society of America	Employment affiliation or facility type, age, gender	Y/Y Regardless of employment or facility type, women's incomes were lower	N/N	N/N
	Kapoor (2017)^39^	573	Academic radiologists at 24 public medical schools	Sex, age, faculty rank, years since residency, clinical trial involvement, NIH funding, total Medicare payments, scientific publications, clinical volume, graduation from a top-20 medical school	Y/N	N/N	N/N
	Nguyen Le (2017)^40^	29,856 in 1990 36,368 in 2000 47,362 in 2010	Physicians from the Integrated Public Use Microdata Series 1990 and 2000 and 2007–2011 ACS (data combined)	Sex, age, race/ethnicity, marital status, number of children, hours worked per week, weeks worked per year, business ownership status	Y/Y After adjustment, the unexplained decrease in women's earnings ranged from 52% to 57%	N/N	N/N
	Jagsi (2016)^41^	2,679	Cardiologists from 161 practices	Age range, gender, race/ethnicity, subspecialty, job characteristics including full-time, work RVUs and new patient office visits, patient care breakdown, geographic region, practice composition and other practice factors, practice compensation model	Y/Y After adjustment, women had lower salaries by $31,749	N/N	N/N
	Jena (2016)^9^	10,241	Academic physicians at 24 public medical schools	Age, sex, experience, specialty, years since residency, faculty rank, NIH funding, clinical trial participation, publication count, medical school attended (top 20 vs. not), Medicare payments, geographic region	Y/Y After adjustment, women had $19,878 lower salaries	N/N	N/N
	Ritter (2016)^42^	1878	Infectious disease physicians, members of the Infectious Diseases Society of America	Practice type, gender, age	Y/Y Gender disparities in income span age ranges and practice types and are greatest for solo/owner/partner physicians	N/N	N/N
	Manahan (2015)^43^	843	Breast surgeons, members of the American Society of Breast Surgeons	Gender, ownership, years of practice, practice type, fellowship training, geographic location, urbanicity, breast surgery case volume and proportion of practice.	Y/Y After adjustment, income was $68,000 lower for women	N/N	N/N
	Spencer (2016)^44^	848	Urologists, members of the American Urologic Association	Age, gender, work hours, call frequency, practice setting and type, fellowship training, Advance Practice Provider employment	Y/Y After adjustment, women had lower compensation	N/N	N/N
	Weaver (2015)^45^	776	Hospitalists who responded to the 2009–2010 Hospital Medicine Physician Worklife Survey	Gender, leadership role, prioritizes substantial pay, pediatric specialty, practice model, practice region, FTE, days per month of clinical work, daily billable encounters	Y/Y After adjustment, women earned $14,581 less	N/N	N/N
	Willett (2015)^46^	241	Internal Medicine program directors, members of the Association of Program Directors in Internal Medicine	Academic rank, career in general internal medicine, age, gender	Y/Y After adjustment, women's salaries were significantly lower	N/N	N/N
	Henderson (2014)^47^	433	Faculty members within four neurological specialties within one health care system (the University of California)	Institution, academic rank, chair status, specialty, Scopus publication count, Scopus h-index	Y/Y Multivariate regression demonstrated women's salaries were 12% lower	N/N	N/N
Neither gender nor race/ethnicity analysis performed	Mead (2020)^48^	1,970	Physicians practicing general orthopedics and seven orthopedic subspecialties who participated in the American Medical Group Association compensation survey	Multivariable analysis not performed—compensation compared against hours worked per week	N/N	N/N	N/N
	Ringel (2019)^49^	358	Endocrinologists, survey of departments via the Association of Endocrine Chiefs and Directors within the Endocrine Society	Multivariable analysis not performed—compensation compared by academic rank, academic track, leadership position (presented separately)	N/N	N/N	N/N
	Chunn (2020)^50^	4,830	Cardiologists in the MedAxiom Annual Survey 2010–2014	Age category, clinical productivity, ownership model, year of survey, compensation method, subspecialty, employment status, days worked, geographic area	N/N	N/N	N/N
	Eltorai (2018)^51^	Not given	Mean data from 37 specialties, data from the American Medical Colleges Careers in Medicine website	Specialty, hours worked	N/N	N/N	N/N
	Mrak (2018)^52^	168	Academic pathologists from 43 departments, survey sent through the Association of Pathology Chairs	Terminal degree(s) with academic rank presented separately from subspecialty with work RVUs	N/N	N/N	N/N
	Prakash (2017)^53^	Not given	Vascular surgeons whose salary data were contained in the Association of American Medical Colleges and Medical Group Management Association databases	Academic vs. private practice, time	N/N	N/N	N/N
	Fijalkowski (2013)^54^	433	Academic physicians in four specialties in the University of California system	Specialty, institution, ranking, sex, number of publications, h-index	N/N	N/N	N/N
	Slakey (2013)^55^	72	U.S. surgery department chairs	Multivariable analysis not performed—Compensation compared by age, additional degree, specialty, location, contract, tenure, clinical hours, program director status, fellowship training separately	N/N	N/N	N/N

ACS, American Community Survey; FTE, full-time equivalent; HSC, Health System Change; NIH, National Institutes of Health; RVUs, relative value units.

Some studies reported gender, race, or ethnicity within general demographic information on participants or used these data as a confounding variable for adjustment in the analysis. Only those studies that reported the impact of each variable on compensation are listed in Table 1 as having analyzed/studied that variable. Table 2 lists the gender, racial, and ethnic breakdown for each study. For studies that reported these categories as percentages, the numbers are noted to be approximate.

**Table 2.** Gender and Racial Breakdown Within Physician Compensation Studies

Study references First author name (year)	No. of participants	Women, *n*	American Indian or Alaskan Native, *n*	Asian, *n*	Black or African American, *n*	Native Hawaiian or Pacific Islander, *n*	Two or more races, *n*	White, *n*	Unknown race or other, *n*	URM^a^, *n*	Other non-URM^a^, *n*	Hispanic, *n*
Hayes (2020)^15^	2,845	861	11	469	57		22	2,120^a^	3			163
Lo Sasso (2020)^6^	16,047	7,005		∼5,182^a^	∼1,103^a^			∼7,466^a^	∼1,278			∼1,199
Langer (2019)	41,396	∼8,859	∼166	∼5,299	∼1,532			∼33,241	∼1,366			∼2,111
Pallant (2019)	149	82		17	7			115^a^				6
Apaydin (2018)^11^	439	176	3	59	9	2		345	4			15
Read (2018)	374	120						125				
Madsen (2017)^19^	1,371	447		98	54			1,066^a^	153			40
Freund (2016)^10^	490	239						429^a^				
Ly (2016)^12^	61,327 from ACS survey 17,583 in HSC survey	16,416 4,222			2,950^a^ 860^a^			58,377^a^ 16,723^a^				
Jagsi (2013)^8^	1,275	419		250	26			688	48			
Seabury (2013)	6,258	1,964										
Rosenthal (2017)	157											
Marcelin (2019)^16^	2,075			333	75			1,401	85			181
Kaplan (2018)	604	309						529^a^		47	28	
Lin (2016)	26 in 2004, 38 in 2009, 54 in 2014	2 in 2004, 11 in 2009, 15 in 2014	Multivariable analysis not performed							2 in 2004, 4 in 2014	22 in 2004, 47 in 2014	
Cheng (2020)	72	35										
Gambhir (2020)	170	50										
Pelley (2020)	Number not given											
Sangji (2020)	461	105	0	29	20		10	383	12			7
Shah (2020)	366	129	5	93	10			197	32			29
Winkelman (2020)	89	53										
Dermody (2019)^14^	260	63										
Horowitz (2019)	366	168		59	15			252	12			19
Wiler (2019)	7,102	2,412		∼284	∼283			∼5,912				
Burns (2018)	97	37										
Hoops (2018)	86	24										
Morris (2018)	44	11										
Trotman (2018)	2,504	∼1,002		∼351	∼75			∼1,502				∼200
Kapoor (2017)	573	171										
Nguyen Le (2017)	29,856 in 1990 36,368 in 2000 47,362 in 2010	6,210 in 1990 9,689 in 2000 15,551 in 2010			∼922 in 1990 ∼1,565 in 2000 ∼1,962 in 2010			∼25,439 in 1990 ∼28,402 in 2000 ∼35,820 in 2010	∼3,466 in 1990 ∼6,403 in 2000 ∼9,581 in 2010			
Jagsi (2016)	2,679	229	1	75	31	4		1,036	40			73
Jena (2016)^9^	10,241	3,549										
Ritter (2016)	1,878	∼751										
Manahan (2015)	843	542										
Spencer (2015)	848	73										
Weaver (2015)	776	263										
Willett (2015)	241	72										
Henderson (2014)	433	98										
Mead (2020)	1,958											
Ringel (2019)	358											
Chunn (2018)	4,830											
Eltorai (2018)												
Mrak (2018)	168											
Prakash (2017)	Not given											
Fijalkowski (2013)	433											
Slakey (2013)	72											

^a^ Category specifically indicated as non-Hispanic.

URM, underrepresented minority.

**FIG. 2.**
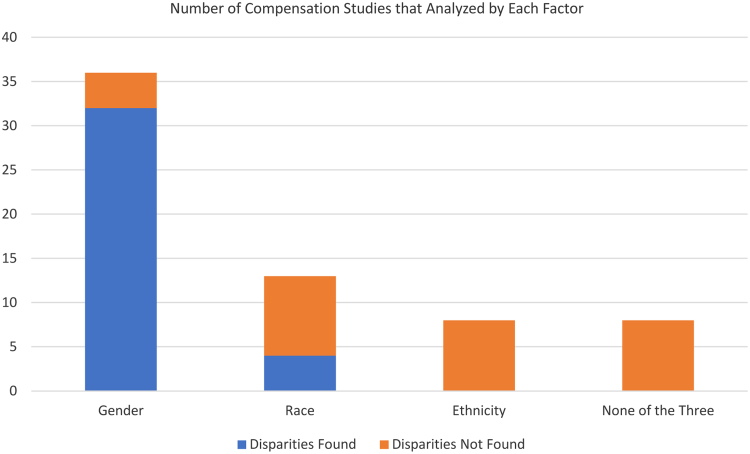
A total of 88.9% of the studies that analyzed by gender found disparities in compensation. A total of 30.7% of the studies that analyzed by race and 0% of the studies that analyzed by ethnicity found disparities.

Of the 36 data sets for which compensation was analyzed by gender, 32 found gender-based compensation disparities (Table 1 and Fig. 2). In contrast, 13 data sets were analyzed by race and 4 found race-based compensation disparities. For ethnicity, zero out of eight data sets showed differences between ethnic groups (Fig. 2). The median sample size for the data sets that were analyzed by race and ethnicity was 1,012 and 987.5. The four data sets that revealed differences in compensation by race had four of the five largest sample sizes in the group of 13 at 61,327, 41,396, 17,583, and 2,075.

## Discussion

In this report, we found that the majority of data sets on physician compensation focus on women and most of these (88.9%) had documented disparities. A smaller number of data sets considered race and/or ethnicity, and of these, four (30.8%) had documented disparities by race.

Our findings are consistent with other reports on gender-related disparities in compensation for physicians.^2,5^ Pay gaps begin early in a physician's career^6^ and persist into the highest echelons of academia.^7^ Documented disparities exist even after accounting for confounding variables, such as years of experience, academic rank, and specialty, among others (Table 1).^8–12^ A recent study showed that research on pay disparities is primarily conducted by women and the majority of this work is unfunded.^13^ Some of the institutions that did not show pay disparities in our study (Table 1) were based on a regimented/formulaic model of compensation,^14,15^ which is one possible approach to address this problem.

Larger studies have reported compensation disparities based on race and/or ethnicity. A 2019 study by Medscape on 19,328 U.S. physicians found that Caucasian physicians receive the highest compensation and African American physicians the lowest.^4^ This racial disparity persisted after adjusting for specialty.^4^ Ly et al. compared the income of White and Black physicians within two large data sets and found that White men made significantly higher compensation than Black men and that, while women physicians made significantly lower income than men physicians, there was no statistically significant difference in compensation for White compared with Black women.^12^ This finding was the same for both data sets they considered.^12^

Marcelin et al. analyzed unadjusted data from a national society report on compensation and found lower compensation for African American physicians within the society.^16^ The authors mentioned that the relatively small number of physicians from underrepresented racial or ethnic groups limited the analysis^16^—a common problem for many compensation studies and databases.

Disparities in rates of promotion can compound compensation disparities. Promotional gaps exist for women physicians.^17^ These are present after adjusting for age, experience, research productivity, and other factors.^18^ Studies have also shown differences in academic rank based on race.^19^ Multivariable compensation studies often adjust for academic rank since compensation is expected to be higher with ascending rank. It is therefore important to consider the additive effect promotional disparities can have on differences in compensation.

### Limitations

This study is limited to articles published in the English language and reported in PubMed, as well as by the search terms used to discover these articles.

## Conclusion

In conclusion, the majority of reports on physician compensation analyzed for and discovered disparities based on gender. A minority of compensation articles considered disparities based on race and/or ethnicity and this analysis was often limited by a small sample size. Disparities in compensation for racial/ethnic minority groups are understudied and further research is needed.

## Abbreviations Used

ACS: American Community Survey
FTE: full-time equivalent
HSC: Health System Change
NIH: National Institutes of Health
RVUs: relative value units
URM: underrepresented minority

## References

[1] Seyfarth. 50 state pay equity desktop reference. 2021. Available at https://www.seyfarth.com/images/content/7/2/v2/72563/50-State-Pay-Equity-Desktop-Reference-RPT-DIGITAL-M21.pdf Accessed July 4, 2021.

[2] Hoff T, Lee DR. The gender pay gap in medicine: a systematic review. Health Care Manage Rev. 2021. DOI: 10.1097/HMR.0000000000000290 33534271

[3] Silver JK. Understanding and addressing gender equity for women in neurology. Neurology. 2019;93:538–549. 31366723 10.1212/WNL.0000000000008022

[4] Medscape. Physician Compensation Report 2019. Available at https://www.medscape.com/slideshow/2019-compensation-overview-6011286 Accessed July 4, 2021.

[5] Lyons NB, Bernardi K, Olavarria OA, et al. Gender disparity among American medicine and surgery physicians: A systematic review. Am J Med Sci. 2021;361:151–168. 33526213 10.1016/j.amjms.2020.10.017

[6] Lo Sasso AT, Armstrong D, Forte G, et al. Differences in starting pay for male and female physicians persists; explanations for the gender gap remain elusive. Health Aff (Millwood). 2020;39:256–263. 31967925 10.1377/hlthaff.2019.00664

[7] Mensah M, Beeler W, Rostenstein L, et al. Sex differences in salaries of department chairs at public medical schools. JAMA Int Med. 2020;180:789–792. 10.1001/jamainternmed.2019.7540 PMC7052792 32119059

[8] Jagsi R, Griffith KA, Stewart A, et al. Gender differences in salary in a recent cohort of early-career physician-researchers. Acad Med. 2013;88:1689–1699. 24072109 10.1097/ACM.0b013e3182a71519 PMC3816636

[9] Jena AB, Olenski AR, Blumenthal DM. Sex differences in physician salary in US public medical schools. JAMA Intern Med. 2016;176:1294–1304. 27400435 10.1001/jamainternmed.2016.3284 PMC5558151

[10] Freund KM, Raj A, Kaplan SE, et al. Inequities in academic compensation by gender: a follow-up to the national faculty survey cohort study. Acad Med. 2016;91:1068–1073. 27276007 10.1097/ACM.0000000000001250 PMC4965349

[11] Apaydin EA, Chen PGC, Friedberg MW. Differences in physician income by gender in a multiregion study. J Gen Intern Med. 2018;33:1579–1581. 10.1007/s11606-018-4462-2 PMC6109018 29752581

[12] Ly DP, Seabury SA, Jena AB. Differences in incomes of physicians in the United States by race and sex: observational study. BMJ. 2016;353:i2923. 27268490 10.1136/bmj.i2923 PMC4897176

[13] Larson AR, Cawcutt KA, Englander MJ, et al. Representation of women in authorship and dissemination of analyses of physician compensation. JAMA Netw Open. 2020;3:e201330. 32196102 10.1001/jamanetworkopen.2020.1330 PMC7084168

[14] Dermody SM, Litvack JR, Randall JA, et al. Compensation of otolaryngologists in the Veterans Health Administration: is there a gender gap? Laryngoscope. 2019;129:113–118. 30152025 10.1002/lary.27311

[15] Hayes SN, Noseworthy JH, Farrugia G. A structured compensation plan results in equitable physician compensation: a single-center analysis. Mayo Clin Proc. 2020;95:35–43. 31902427 10.1016/j.mayocp.2019.09.022

[16] Marcelin JR, Bares SH, Fadul N. Improved infectious diseases physician compensation but continued disparities for women and underrepresented minorities. Open Forum Infect Dis. 2019;6:ofz042. 30815507 10.1093/ofid/ofz042 PMC6386799

[17] Nocco SE, Larson AR. Promotion of women physicians in academic medicine. J Womens Health (Larchmt). 2021;30:864–871. 32407186 10.1089/jwh.2019.7992

[18] Jena AB, Khullar D, Ho O, et al. Sex Differences in academic rank in US medical schools in 2014. JAMA. 2015;314:1149–1158. 26372584 10.1001/jama.2015.10680 PMC4665995

[19] Madsen TE, Linden JA, Rounds K, et al. Current status of gender and racial/ethnic disparities among academic emergency medicine physicians. Acad Emerg Med. 2017;24:1182–1192. 28779488 10.1111/acem.13269

[20] Langer AL, Laugesen M. Billing codes determine lower physician income for primary care and non-procedural specialties. Forum Health Econ Policy. 2019. DOI: 10.1515/fhep-2019-0009. 31837254

[21] Pallant A, Hudson SM, Ben-Isaac E. Satisfaction, salaries, and sustainability: Results of a national survey of pediatric program directors. Acad Pediatr. 2019;19:11–17. 30287393 10.1016/j.acap.2018.09.015

[22] Read S, Butkus R, Weissman A, Moyer DV. Compensation disparities by gender in internal medicine. Ann Intern Med. 2018;169:658–661. 30083760 10.7326/M18-0693

[23] Seabury SA, Chandra A, Jena AB. Trends in the earnings of male and female health care professionals in the United States, 1987 to 2010. JAMA Intern Med. 2013;173:1748–1750. 10.1001/jamainternmed.2013.8519 23999898

[24] Rosenthal LJ, Sabuco JJ. Salaries in psychosomatic medicine: A cross-sectional survey of practicing physicians. Psychosomatics. 2017;58:92–94. 28010751 10.1016/j.psym.2016.07.003

[25] Kaplan SE, Raj A, Carr PL, Terrin N, Breeze JL, Freund KM. Race/ethnicity and success in academic medicine: Findings from a longitudinal multi-institutional study. Acad Med. 2018;93:616–622. 29068820 10.1097/ACM.0000000000001968 PMC5916738

[26] Lin SY, Francis HW, Minor LB, Eisele DW. Faculty diversity and inclusion program outcomes at an academic otolaryngology department. Laryngoscope. 2016;126:352–356. 26153871 10.1002/lary.25455

[27] Cheng Y, Mohanty AF, Ogunyemi OI, Smith CA, Leroy G, Zeng QT. 2018 Salary survey of AMIA members: Factors associated with higher salaries. AMIA Annu Symp Proc. 2020;2019:275–284. 32308820 PMC7153054

[28] Gambhir S, Daly SC, Elfenbein D, Sheehan B, Maithel S, Smith M, Nguyen NT. The effect of transparency on the gender-based compensation gap in surgical disciplines within a large academic healthcare system. Surg Endosc. 2021;35:2607–2612. 32488656 10.1007/s00464-020-07679-1

[29] Pelley E, Carnes M. When a specialty becomes "Women's work": Trends in and implications of specialty gender segregation in medicine. Acad Med. 2020;95:1499–1506. 32590470 10.1097/ACM.0000000000003555 PMC7541620

[30] Sangji NF, Fuentes E, Donelan K, Cropano C, King D. Gender disparity in trauma surgery: Compensation, practice patterns, personal life, and wellness. J Surg Res. 2020;250:179–187. 32070837 10.1016/j.jss.2019.12.048

[31] Shah SO, Latorre JGS. Neurocritical care society survey working group. 2019 Neurocritical care survey: Physician compensation, unit staffing and structure. Neurocrit Care. 2020;33:303–307. 32632907 10.1007/s12028-020-01032-z

[32] Winkelman WD, Jaresova A, Hacker MR, Richardson ML. Salary disparities in academic urogynecology: Despite increased transparency, men still earn more than women. South Med J. 2020;113:341–344. 32617594 10.14423/SMJ.0000000000001119 PMC10152895

[33] Horowitz E, Feldman HA, Savich R. Neonatologist salary: factors, equity and gender. J Perinatol. 2019;39:359–365. 30617285 10.1038/s41372-018-0304-7

[34] Wiler JL, Rounds K, McGowan B, Baird J. Continuation of gender disparities in pay among academic emergency medicine physicians. Acad Emerg Med. 2019;26:286–292. 30664286 10.1111/acem.13694

[35] Burns KH, Borowitz MJ, Carroll KC, Gocke CD, Hooper JE, Amukele T, Tobian AAR, Valentine A, Kahl R, Rodas-Eral V, Boitnott JK, Jackson JB, Sanfilippo F, Hruban RH. The evolution of earned, transparent, and quantifiable faculty salary compensation: The Johns Hopkins pathology experience. Acad Pathol. 2018. DOI: 10.1177/2374289518777463. PMC6024278 29978019

[36] Hoops HE, Brasel KJ, Dewey E, Rodgers S, Merrill J, Hunter JG, Azarow KS. Analysis of gender-based differences in surgery faculty compensation, promotion, and retention: Establishing equity. Ann Surg. 2018;268:479-487. 30063494 10.1097/SLA.0000000000002920

[37] Morris M, Chen H, Heslin MJ, Krontiras H. A structured compensation plan improves but does not erase the sex pay gap in surgery. Ann Surg. 2018;268:442–448. 29979249 10.1097/SLA.0000000000002928

[38] Trotman R, Kim AI, MacIntyre AT, Ritter JT, Malani AN. 2017 Infectious Diseases Society of America Physician Compensation Survey: Results and Analysis. Open Forum Infect Dis. 2018;5:ofy309. DOI: 10.1093/ofid/ofy309. 30555851 PMC6288767

[39] Kapoor N, Blumenthal DM, Smith SE, Ip IK, Khorasani R. Sex differences in radiologist salary in U.S. public medical schools. AJR Am J Roentgenol. 2017;209:953–958. 28871808 10.2214/AJR.17.18256

[40] Nguyen Le TA, Lo Sasso AT, Vujicic M. Trends in the earnings gender gap among dentists, physicians, and lawyers. J Am Dent Assoc. 2017;148:257–262. 28238360 10.1016/j.adaj.2017.01.005

[41] Jagsi R, Biga C, Poppas A, Rodgers GP, Walsh MN, White PJ, McKendry C, Sasson J, Schulte PJ, Douglas PS. Work activities and compensation of male and female cardiologists. J Am Coll Cardiol. 2016;67:529–541. 26560679 10.1016/j.jacc.2015.10.038 PMC4962867

[42] Ritter JT, Lynch JB 3rd, MacIntyre AT, Trotman R. Infectious diseases physician compensation: An improved perspective. Open Forum Infect Dis. 2016;3:ofw083. DOI: 10.1093/ofid/ofw083. 27419159 PMC4943553

[43] Manahan E, Wang L, Chen S, Dickson-Witmer D, Zhu J, Holmes D, Kass R. What is a breast surgeon worth? A salary survey of the american society of breast surgeons. Ann Surg Oncol. 2015;22:3257–3263. 26202565 10.1245/s10434-015-4720-z

[44] Spencer ES, Deal AM, Pruthi NR, Gonzalez CM, Kirby EW, Langston J, McKenna PH, McKibben MJ, Nielsen ME, Raynor MC, Wallen EM, Woods ME, Pruthi RS, Smith AB. Gender differences in compensation, job satisfaction and other practice patterns in urology. J Urol. 2016;195:450–455. 26384452 10.1016/j.juro.2015.08.100 PMC5004345

[45] Weaver AC, Wetterneck TB, Whelan CT, Hinami K. A matter of priorities? Exploring the persistent gender pay gap in hospital medicine. J Hosp Med. 2015;10:486–490. 26122400 10.1002/jhm.2400

[46] Willett LL, Halvorsen AJ, McDonald FS, Chaudhry SI, Arora VM. Gender differences in salary of internal medicine residency directors: a national survey. Am J Med. 2015;128:659–665. 25731136 10.1016/j.amjmed.2015.02.002

[47] Henderson MT, Fijalkowski N, Wang SK, Malenfort M, Zheng LL, Ratliff J, Moshfeghi AA, Moshfeghi DM. Gender differences in compensation in academic medicine: the results from four neurological specialties within the University of California Healthcare System. Scientometrics. 2014;100:297–306.

[48] Mead M, Atkinson T, Srivastava A, Walter N. The return on investment of orthopaedic fellowship training: A ten-year update. J Am Acad Orthop Surg. 2020;28:e524–e531. DOI: 10.5435/JAAOS-D-19-00276. 31688369

[49] Ringel MD, Murphy EJ, Hammes SR. Compensation, productivity, and other demographics of academic divisions of endocrinology, diabetes, and metabolism. J Endocr Soc. 2019;3:1485–1502. 31384713 10.1210/js.2019-00095 PMC6676069

[50] Chunn VM, Sen B, O'Connor SJ, Jessee WF, Sasson J, Landry AY. Integration of cardiologists with hospitals: Effects on physician compensation and productivity. Health Care Manage Rev. 2020;45:342–352. 30299382 10.1097/HMR.0000000000000223

[51] Eltorai AEM, Eltorai AS, Fuentes C, Durand WM, Daniels AH, Ali S. Financial implications of physician specialty choice. R I Med J (2013). 2018;101:50–55. 30278604

[52] Mrak RE, Parslow TG, Ducatman BS. Benchmarking subspecialty practice in academic anatomic pathology: The 2017 association of pathology chairs survey. Acad Pathol. 2018. DOI: 10.1177/2374289518798556. PMC6178122 30327790

[53] Prakash S, Satiani B. Analysis of compensation disparities between junior academic and private practice vascular surgeons. Ann Vasc Surg. 2017;39:236–241. 27554692 10.1016/j.avsg.2016.05.127

[54] Fijalkowski N, Zheng LL, Henderson MT, Moshfeghi AA, Maltenfort M, Moshfeghi DM. Academic productivity and its relationship to physician salaries in the University of California Healthcare System. South Med J. 2013;106:415–421. 23820322 10.1097/SMJ.0b013e31829b9dae

[55] Slakey DP, Korndorffer JR, Long KN, Clark T, Hidalgo M. The modern surgery department chairman: the job description as identified by chairmen. JAMA Surg. 2013;148:511–515. 23754568 10.1001/jamasurg.2013.1230

